# Evaluation of Trunk Mobility in Spanish High-Level National Rhythmic Gymnastics Athletes with Low Back Pain: A Randomized Controlled Trial Comparing the Mézières Method and Isostretching Postures

**DOI:** 10.3390/jcm14082584

**Published:** 2025-04-09

**Authors:** Orges Lena, Erda Qorri, Juan Martínez-Fuentes, Jasemin Todri

**Affiliations:** 1 ÍTEM-Innovation in Manual and Physical Therapies-Research Group, Physiotherapy Department, UCAM Universidad Católica San Antonio de Murcia, Campus de los Jerónimos, Nº 135 Guadalupe, 30107 Murcia, Spain; lorges@ucam.edu; 2 Department of Dentistry, Faculty of Medical Sciences, Albanian University, 1001 Tirana, Albania; e.qorri@albanianuniversity.edu.al; 3 Physiotherapy Department, UCAM Universidad Católica San Antonio de Murcia, Campus de los Jerónimos, Nº 135 Guadalupe, 30107 Murcia, Spain; jmfuentes@ucam.edu

**Keywords:** Mézières method, isostretching postures, rhythmic gymnastics, low back pain, high-level national athletes, Baiobit sensor

## Abstract

**Background/Objectives:** Postural therapies have emerged as effective non-invasive approaches to managing and preventing LBP in athletes. These therapies focus on correcting muscular imbalances, enhancing body awareness, and promoting proper alignment. Therefore, the objective of this study was to evaluate the effectiveness of implementing the Mézières method and isostretching postures in Spanish high-level national rhythmic gymnasts with low back pain (LBP). Specifically, the study aims to assess the impact of these postural therapies on functional limitations associated with LBP. **Methods:** This study was a parallel group, randomized controlled trial implemented in 17 rhythmic gymnastics athletes with low back pain conducted at baseline, at 2, 4, and 6 weeks, and upon completion of the 12-week intervention period. The outcome measures included pain measurement, the Borg effort scale, and trunk movement as flexion, extension, inclination, and rotation assessed using a wearable device sensor. **Results:** The trial included eight participants in the Mézières group and nine in the isostretching group, with no significant age difference between the groups (*p* = 0.589). Significant differences were found for time (*p* = 0.000) and group (*p* = 0.001), indicating variations in left trunk inclination performance over time and between the groups. Both groups showed increased trunk flexion, with the Mézières group demonstrating higher values at all time points. Right trunk rotation fluctuated, with a notable increase in the Mézières group at 6 weeks. The Mézières group also showed higher left trunk rotation values, peaking at 6 weeks. **Conclusions:** Although the Mézières method showed certain advantages in right trunk rotation and left tilt, the results overall suggest that both approaches are effective in the specific context of this population.

## 1. Introduction

Low back pain (LBP) is a widespread musculoskeletal condition that significantly affects individuals across various populations, including athletes engaged in elite-level sports [1,2]. Among rhythmic gymnasts, LBP is particularly prevalent due to the sport’s unique biomechanical demands, which involve repetitive trunk movements, extreme flexibility, and high-impact landings [3,4]. These factors place considerable stress on the lumbar spine, leading to an increased risk of overuse injuries, postural imbalances, and chronic discomfort [5]. Recent research highlights the importance of addressing LBP in rhythmic gymnasts, not only to optimize performance but also to prevent long-term health consequences [5,6].

The trunk plays a central role in the biomechanical framework of rhythmic gymnastics, acting as a stabilizer and power generator for complex movements. Excessive lumbar extension, frequent twisting, and hyperflexion are common in routines, placing high mechanical loads on the spine and surrounding musculature. Studies have shown that inadequate core stability and improper motor control can exacerbate these loads, increasing the likelihood of LBP [7]. Furthermore, asymmetries in trunk strength and flexibility, often developed due to unilateral training patterns, have been identified as key contributors to LBP in gymnasts [8].

A 2024 study by Deodato et al. explored the relationship between trunk mobility and LBP in rhythmic gymnasts, revealing that restricted or excessive trunk movement patterns correlate strongly with pain severity. The findings underscore the need for interventions that target both mobility and stability to maintain optimal spinal health in this population [9].

Postural therapies have emerged as effective non-invasive approaches to managing and preventing LBP in athletes. These therapies focus on correcting muscular imbalances, enhancing body awareness, and promoting proper alignment [10]. Among the most promising techniques are the Mézières method and isostretching postures, both emphasizing global postural re-education and core strengthening [11,12].

The Mézières method, developed by Françoise Mézières, is based on the principle of restoring postural balance by addressing muscular chains. This method involves specific stretching exercises aimed at elongating shortened muscles, improving joint mobility, and re-establishing proper postural alignment [12]. A fundamental characteristic of the Mézières method is its focus on muscle chain rebalancing, as it considers that any alteration in one segment of the body affects the entire posture. Additionally, it integrates diaphragmatic breathing, which plays a crucial role in promoting relaxation and optimizing postural control. Studies have demonstrated its effectiveness in reducing LBP and enhancing functional performance in athletes [11,12].

In rhythmic gymnastics, the Mézières method can be particularly beneficial due to its emphasis on correcting compensatory movement patterns and promoting symmetrical trunk control. A randomized controlled trial (RCT) indicated that incorporating the Mézières method into the training regimen of gymnasts with LBP resulted in significant reductions in pain intensity and improvements in trunk flexibility and stability [10]. Additionally, research has shown that the Mézières method improves proprioception and spinal mobility, which are critical components for gymnasts who rely on precise motor control [12].

Isostretching is a postural exercise technique that combines isometric contractions with stretching to strengthen deep stabilizing muscles while maintaining proper alignment [13]. Unlike conventional stretching routines, isostretching emphasizes a controlled and progressive approach, targeting flexibility and core stabilization simultaneously [13]. This method has been found to enhance postural control, alleviate spinal stress, and improve breathing coordination, making it particularly suitable for rhythmic gymnasts, who require high levels of flexibility and stability [14]. Although specific studies on isostretching in rhythmic gymnastics are limited, research on similar interventions has demonstrated positive outcomes in reducing LBP and improving postural control. A 2021 study by Prado et al. reported that athletes who participated in a structured isostretching program experienced reduced pain levels and enhanced trunk endurance, underscoring its potential benefits for gymnasts [14].

Therefore, the objective of this study is to evaluate the effectiveness of implementing the Mézières method and isostretching techniques in high-level national rhythmic gymnasts with low back pain. Specifically, the study aims to assess the impact of these postural therapies on functional limitations associated with LBP.

## 2. Methods

### 2.1. Description of Trial Design

This study was a parallel group, randomized controlled trial with a 1:1 allocation ratio. A total of 17 Spanish high-level national rhythmic gymnastics athletes with low back pain were recruited and randomly assigned to either the experimental group (*n* = 8) or the control group (*n* = 9). The intervention lasted for 12 weeks, with participants in both groups attending two weekly sessions. The experimental group received an intervention based on the Mézières method, while the parallel group performed isostretching postures. Assessments were conducted at baseline, at 2, 4, and 6 weeks, and upon completion of the 12-week intervention period. These assessments included measures of the trunk movements as flexion, extension, and rotation. This parallel design ensured a structured comparison between the two groups and allowed for evaluation of the efficacy of the Mézières method in addressing low back pain in high-level national rhythmic gymnasts.

No important changes to the methods after trial commencement were reported. The treatments were free of adverse effects.

### 2.2. Ethics Committee Approval

This study was conducted in accordance with the principles outlined in the Declaration of Helsinki to ensure the ethical treatment and protection of all participants. Prior to the commencement of the study, the research protocol was reviewed and approved by the Catholic University San Antonio of Murcia, Spain, Ethics Committee approbation on 29 October 2021 with ID: CE102105, ensuring compliance with ethical standards for human research. The trial was registered at ClinicalTrials.gov with ID: NCT05149703. All participants, or their legal guardians in the case of minors, provided written informed consent before any data collection began. The consent process included a thorough explanation of the study’s objectives, procedures, potential benefits, and risks. Participants were informed of their right to withdraw from the study at any time without any repercussions or negative impact on their ongoing care or training. This commitment to ethical transparency ensured that all participants were fully aware of their involvement and voluntarily agreed to participate in the trial.

### 2.3. Participants

The participants were Spanish high-level national rhythmic gymnasts aged between 10 and 30 years old who were required to have a confirmed clinical diagnosis of low back pain with ongoing symptomatology at the time of the study, regardless of severity. The pain had to be restricted to the lumbopelvic region. The inclusion criteria required athletes to be actively competing at the highest national level in Spain, specifically participating in Spain’s National Championships. They were also required to be training at a competitive level, with a minimum training load that aligns with high-performance rhythmic gymnastics. Additionally, all participants had to have a confirmed medical diagnosis of low back pain with an underlying cause of musculoskeletal dysfunction (e.g., postural imbalances, muscle tightness, weakness of deep stabilizing muscles) or mechanical stress-related pain (e.g., repetitive lumbar hyperextension, asymmetrical loading due to sport-specific movements). Exclusion criteria included the presence of any systemic or neurological conditions, recent spinal surgery, structural spinal pathologies, including herniated disks, spondylolisthesis, or acute musculoskeletal injuries affecting the lumbar spine, or other musculoskeletal injuries that could interfere with participation in the interventions. Additionally, athletes were excluded if they had received any similar physical therapy treatments targeting low back pain within the three months prior to the study as Pilates, yoga, etc. The data were collected at a training camp for the athletes.

### 2.4. Interventions

#### 2.4.1. Mézières Method Intervention (Experimental Group)

Participants in the experimental group received the Mézières method intervention, which focuses on global postural re-education through the elongation of muscle chains and the correction of postural imbalances. The intervention was delivered by a certified physical therapist trained in the Mézières method [10,15]. Participants attended two sessions per week, each lasting approximately 60 min, over the course of 12 weeks (24 sessions in total). The Mézières session structure was as follow:

(a) Initial evaluation: Each session began with an assessment of the participant’s posture and muscular tension to guide individualized treatment.
(b) Positioning: Participants were placed in specific postures designed to stretch shortened muscle chains, focusing on restoring alignment. Common postures included supine lying with legs extended or flexed at 90 degrees and arms positioned to counterbalance muscular tension.
(c) Breathing integration: Diaphragmatic breathing exercises were incorporated throughout the session to enhance relaxation and promote proper muscle elongation.
(d) Manual techniques: The therapist applied gentle manual stretching and corrections to address specific areas of tension or asymmetry.
(e) Progression: Adjustments were made in subsequent sessions based on the participant’s response to treatment and progress toward achieving postural realignment.

#### 2.4.2. Isostretching Intervention (Parallel Experimental Group)

Participants in the parallel group performed isostretching exercises, which were designed to simulate a placebo treatment. Isostretching combines static postures and mild stretches to promote flexibility and core activation without targeting specific postural corrections [13,14,16]. 

Similarly to the experimental group, participants in the parallel experimental group attended two sessions per week, each lasting approximately 60 min, over the 12-week intervention period (24 sessions in total). The isostretching session structure was as follow:

(a) Warm-up: Sessions began with a light warm-up consisting of general body movements to prepare the participants for stretching.
(b) Stretching postures: Participants performed a series of standard isostretching postures, such as seated or standing positions that incorporated static holds with mild spinal and limb stretches.
(c) Core activation: Exercises included basic isometric holds focusing on engaging the abdominal and back muscles. Movements were performed within the pain-free range and without adjustments targeting postural asymmetry.
(d) Cool-down: Sessions concluded with relaxation exercises, such as slow diaphragmatic breathing, to ensure a calming finish.

Both interventions were conducted in a controlled environment under the direct supervision of the respective therapist. Adherence was monitored by recording attendance, and therapists ensured the correct execution of techniques during each session.

### 2.5. Outcome Measures

The outcome measures included the numeric rating scale (0–10), the modified Borg scale (0–10), and trunk movement as flexion, extension, and rotation, and the movements were assessed using the Baiobit wearable device sensor.

Pain intensity was assessed using the numeric rating scale (NRS), a reliable and validated tool for measuring pain on a 0–10 scale, where higher scores indicate greater pain. It is widely used in both clinical and experimental settings and has demonstrated sensitivity in evaluating treatment effects [17,18].

The modified Borg Scale (MBS) was used for this trial, perceiving exertion that assesses the intensity of physical effort, breathlessness, and fatigue during activity. It is a numerical scale ranging from 0 to 10, where 0 represents “no exertion at all” and 10 indicates “maximal exertion”. The scale is widely used in clinical and research settings to monitor exertion levels in patients with cardiovascular, pulmonary, and musculoskeletal conditions. The modified Borg scale is an adaptation of the original Borg rating of perceived exertion (RPE) scale, which ranged from 6 to 20. This modified version provides a more intuitive and simplified approach for assessing effort and is commonly applied in rehabilitation and exercise prescription (Borg, 1998) [19,20,21].

The range of motion (ROM) measures were chosen to evaluate the functional improvements in trunk mobility, a critical component in the management of low back pain and athletic performance at baseline, 2 weeks, 4 weeks, 6 weeks, and 12 weeks of intervention. Trunk flexion, defined as the forward bending of the trunk, was measured in degrees with participants standing and the sensor positioned at the lumbar spine. Trunk extension, the backward bending of the trunk, was similarly recorded, ensuring movements were performed safely and without pain. Trunk rotation, measured bilaterally as the rotational movement to the left and right, was also assessed in degrees with the sensor capturing the maximum range of motion in each direction. These repeated measures allowed for the detailed tracking of trunk mobility improvements over time.

The Baiobit is a wearable motion analysis system developed by Rivelo S.r.l., an Italian company based in Garbagnate Milanese, Italy. Introduced in 2020, Baiobit is a lightweight and user-friendly medical device designed for precise and rapid movement assessments. The system includes a single wearable motion sensor (which utilizes accelerometers, gyroscopes, and magnetometers to capture real-time movement data with high accuracy) and three adjustable belts to accommodate various body segments, allowing for the comprehensive analysis of joint range of motion, postural stability, and gait patterns. It is paired with a user-friendly software interface that processes and analyzes biomechanical parameters, including range of motion (ROM), postural stability, and gait analysis.

This technology supports professionals in evaluating motor functions and guiding therapeutic exercises, providing real-time data and comprehensive analysis to tailor interventions effectively.

Recent studies have evaluated its validity and reliability in measuring these movements, showing a high correlation with optoelectronic systems, with inter-instrument correlation coefficients ranging from 0.90 to 0.99, indicating strong agreement in measuring trunk movements [22].

When compared to standard measurement tools like goniometers, the Baiobit sensor demonstrates minimal mean differences, typically less than 2 degrees, supporting its accuracy in capturing trunk range of motion (ROM). The sensor exhibits an intra-instrument coefficient of variation of ≤2.5%, reflecting consistent performance across repeated measurements. Studies report high intraclass correlation coefficients (ICCs) for repeated assessments, with values exceeding 0.90, indicating excellent reliability over time [23,24].

This wearable sensor operates with an accelerometer frequency bandwidth ranging from 4 to 1000 Hz and a gyroscope bandwidth from 4 to 8000 Hz, allowing for precise motion capture [24].

It is also a valid and reliable tool for assessing trunk movements, offering high correlation with gold-standard measurement systems and consistent performance across repeated assessments. Its technical specifications enable precise motion analysis, making it suitable for both clinical and research applications [25].

All assessments were conducted in a controlled environment, supervised by trained evaluators to ensure the accuracy of measurements and participant safety. Participants completed a standardized warm-up before testing to reduce the risk of injury and ensure consistency. Three repetitions of each movement were performed for all trunk movements, and the average value was used for analysis.

**Figure 1:**
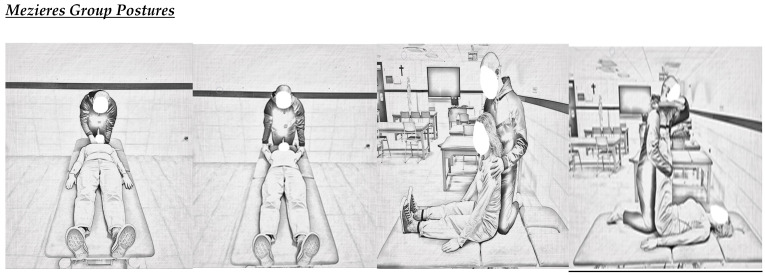

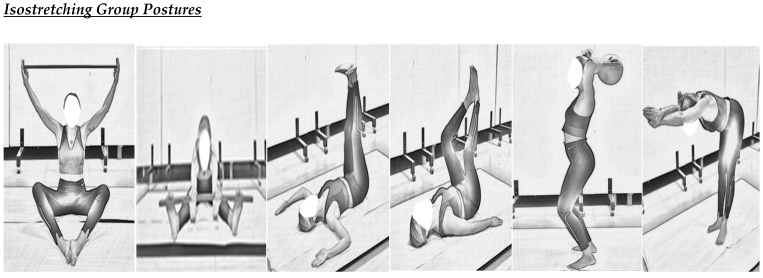
Postural implementation illustrated protocol.

Figure 1 displays the illustrated postures of Mezieres and isostretching groups.

### 2.6. Sample Size

The sample size for this study was determined based on the specific characteristics of the population, feasibility, and statistical considerations, referring to the target population of Spanish high-level national rhythmic gymnasts with low back pain, a highly specialized group, which limited recruitment possibilities. Also, the exploratory trial design aimed to evaluate preliminary outcomes, making a smaller sample size appropriate. As per power analysis, a priori calculations performed with G*Power Program, assuming a medium-to-large effect size (Cohen’s *d* = 0.8), determined that 16 participants (8 per group) would provide 80% power with an alpha level of 0.05. A 1:1 allocation ratio (8 experimental, 9 parallel experimental) ensured balance and accounted for potential dropouts. The sample size was sufficient for assessing the feasibility and preliminary effectiveness of the interventions in this specialized population.

### 2.7. Randomization

The allocation sequence for this study was created using a computerized random number generator to guarantee the impartial and unpredictable assignment of participants. The sequence allocated individuals in a 1:1 ratio to either the experimental group (Mézières method) or the parallel experimental group (isostretching). To ensure group balance, stratification by key factors, such as age and baseline severity of low back pain, was considered to reduce potential confounding variables. The allocation process was concealed by an adaptive biased-coin design, preventing prior knowledge of group assignments, and ensuring proper allocation concealment.

The random allocation sequence was generated by an independent researcher who was not involved in participant recruitment or intervention administration. This ensured that the sequence was unbiased and unpredictable. Participants were enrolled by a study coordinator responsible for explaining the study, obtaining written informed consent, and verifying eligibility criteria. The coordinator was blinded to the allocation sequence. Participants were assigned to their respective intervention groups by a third researcher who accessed the concealed allocation sequence. This third researcher was not involved in the intervention delivery or outcome assessments, maintaining blinding for other study personnel. This separation of roles ensured strict adherence to the study protocol and minimized the risk of allocation bias.

### 2.8. Blinding

Participants were partially blinded, as they were aware they were receiving an intervention (Mézières method or isostretching), but they were not informed of the specific hypotheses, or which intervention was considered the experimental treatment versus the placebo. The care providers delivering the interventions were not blinded, as the techniques (Mézières and isostretching) required specific training and a clear understanding of the respective protocols. Those assessing outcomes (e.g., range of motion measurements via the Baiobit wearable sensor) were blinded to group assignments. They were not informed of which participants belonged to the experimental or parallel groups. The outcome assessments relied on objective data collected by the sensor, further reducing the potential for bias. To maintain blinding of the assessors, participants were instructed not to disclose their group allocation or details of their intervention during assessments. Additionally, group allocation information was securely stored and only accessible to the researcher responsible for managing assignments. This approach ensured a high level of blinding where feasible, particularly for those assessing outcomes, to enhance the study’s internal validity.

### 2.9. Statistics

Means and standard deviations (SDs) were calculated for continuous variables as the range of motion in trunk flexion, extension, and rotation to summarize the baseline characteristics and outcomes for each group. Independent *t*-tests were used to compare the continuous outcome variables as the change in trunk movement between the experimental group (Mézières method) and the parallel group (isostretching) at each time point (baseline, 2 weeks, 4 weeks, 6 weeks, and 12 weeks). Analysis of covariance (ANCOVA) was used to adjust for baseline values of the outcomes, which helps control for any initial differences between groups that might influence the results. Two-way repeated measures ANCOVA were also performed to verify the difference between groups over time, groups, and groups ∗ time. A *p*-value of <0.05 was considered statistically significant for all comparisons. Confidence intervals of 95% were also provided for the statistical performance. All statistical analyses were performed using SPSS (Statistical Package for the Social Sciences, version 25, IBM Corporation, Armonk, NY, USA) software. 

## 3. Results

Out of 35 assessed participants, 15 were excluded for not meeting the inclusion criteria, leaving 20 gymnasts who were randomized into two intervention groups: Mezieres (*n* = 10) and isostretching (*n* = 10).

In the Mezieres group, eight participants received the allocated intervention, while two did not participate due to a team change. In the isostretching group, nine participants received the allocated intervention, and one dropped out due to leaving the team.

**Figure 2:**
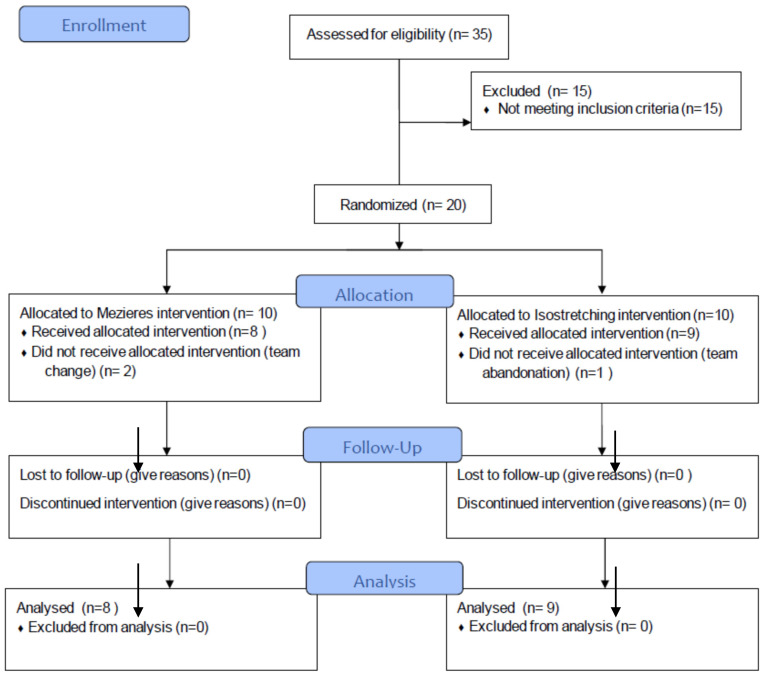
Participant’s flowchart.

There were no losses to follow-up or discontinuations in either group. Ultimately, eight participants in the Mezieres group and nine in the isostretching group were analyzed, with no exclusions from the final analysis (Figure 2).

The average age was 13.38 ± 0.92 years in the Mézières group and 14.33 ± 4.82 years in the isostretching group, with no significant difference between the groups (*p* = 0.589). Participants in the Mézières group had a mean height of 157.25 ± 12.85 cm, compared to 155.11 ± 6.64 cm in the isostretching group (*p* = 0.667).

The baseline NRS scores indicate similar pain levels between the Mezieres (4.94 ± 1.21 cm) and isostretching (5.72 ± 1.48 cm) groups, with no significant difference (*p* = 0.254). Likewise, perceived exertion on the Borg scale was comparable between the Mezieres (4.38 ± 1.06 cm) and isostretching (4.00 ± 0.87 cm) groups, with no significant difference (*p* = 0.298).

Table 1 displays the two study groups characteristics.

The results indicate a significant reduction in pain (NRS) over time (*p* = 0.005), with the isostretching group experiencing greater pain relief (*p* = 0.000) and distinct pain reduction trends between groups (*p* = 0.026).

Trunk flexion and extension improved over time in both groups without significant differences between them.

Right rotation showed a significant interaction effect (*p* = 0.023), suggesting different progression patterns between groups. Concretely, the Mézières group displayed a notable increase at the 6-week mark.

The Mézières group exhibited higher left rotation values at most time points, reaching a peak at 6 weeks. Despite this, no significant differences were identified for time (*p* = 0.441), group (*p* = 0.431), or the group ∗ time interaction (*p* = 0.059) (Table 2). Additionally, left inclination improved significantly over time (*p* = 0.000), with one group demonstrating greater enhancements (*p* = 0.001) (Table 2).

**Figure 3:**
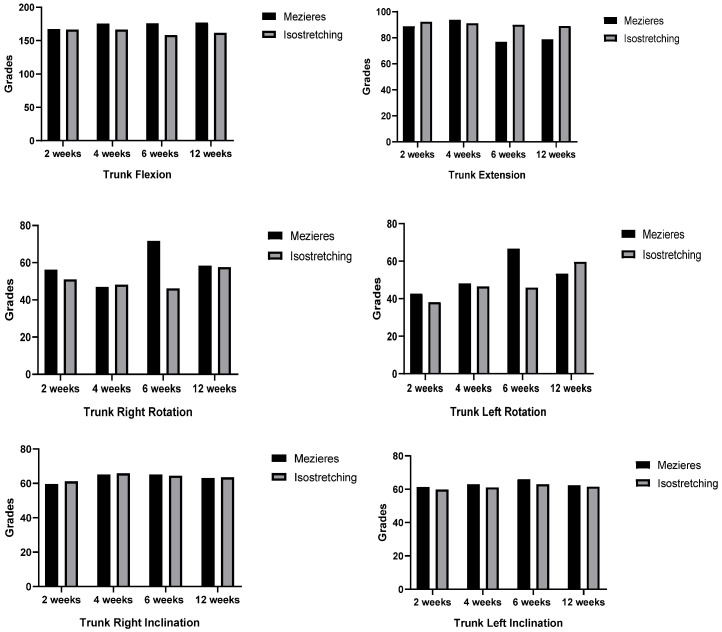
Two-way ANCOVA analysis of trunk mobility changes over time.

Two-way ANCOVA analysis revealed a significant difference in right rotation at 6 weeks (*p* = 0.006), with the Mézières group having a higher mean (71.75) than the isostretching group (46.22). A similar significant difference was found for left rotation at 6 weeks (*p* = 0.015), where the Mézières group had a higher mean (66.75) compared to the isostretching group (46.00). For the other trunk movements, no significant differences were observed between the Mézières and isostretching groups at any time point (all *p*-values > 0.05) (Figure 3).

## 4. Discussion

This research explored the effectiveness of the Mézières method and isostretching in high-level national rhythmic gymnasts with low back pain, revealing differentiated findings in trunk mobility. A key result was the significant group ∗ time interaction (*p* = 0.023), which suggests that the changes in right trunk rotation over time varied between the Mézières and isostretching groups. Notably, the Mézières group showed a sharp increase in right rotation at 6 weeks compared to the isostretching group, indicating differential responses to the interventions.

Concretely, the Mezieres group achieved a higher mean (71.75°) than the isostretching group (46.22°), likely due to its focus on global postural re-education and deep muscle activation. Rhythmic gymnasts, known for their extreme flexibility and asymmetric movements, benefit differently from various training methods, leading to variations in effectiveness.

This finding suggests that the Mézières method might have a more pronounced effect on improving trunk rotation within a specific time frame. This result aligns with previous studies that highlight the benefits of postural therapies in improving mobility, as reflected in the study by Lena et al. where an improvement in back flexibility and pain reduction was observed in rhythmic gymnastics athletes using the Mézières method [10], as well as in the study by Alfonso-Mora et al. which reported similar results in an adult population with low back pain [15].

Furthermore, significant differences for time (*p* = 0.000) and group (*p* = 0.001) indicate that the overall performance in left trunk inclination varied across time points and between the two groups.

These findings reflect the specific adaptations of high-level national rhythmic gymnasts, including their repetitive training patterns, asymmetrical movement tendencies, and pre-existing hypermobility, which influence how they respond to flexibility and mobility interventions.

The Mézières group consistently showed slightly better results, highlighting its potential advantage in improving left inclination, which could be related to better postural control and trunk alignment, core elements of the Mézières method. This improvement in lateral tilt may be relevant in gymnasts, who require precise control of their body in various positions and movements, as some studies point out regarding the importance of lumbopelvic muscle control [26], and especially in the performance of rhythmic gymnasts [9].

Despite these differences in right rotation and left tilt, it is important to note that, in many other measures, the differences between the groups did not reach statistical significance, although the Mézières group showed a trend towards obtaining slightly higher average values. This lack of significant differences may be due to the fact that, although with different approaches, both interventions share several aspects that may have contributed to similar results in the other trunk movements. As mentioned earlier, both treatments shared the same duration and frequency (2 weekly sessions for 12 weeks), an individualized approach to the needs of each gymnast, the common goal of improving flexibility and mobility, and supervision by qualified professionals. These similarities in treatment structure may have mitigated the differences between the interventions, as seen in studies exploring the effectiveness of other treatments for low back pain where individualized attention and constant monitoring are important [15].

The presence of significant differences between Mézières and isostretching, mainly in right and left rotation at 6 weeks as evidenced in the multivariate ANCOVA analysis, suggests that the treatments may have differentiated effects on specific trunk movements, but not in other mobility movements. These findings highlight the importance of considering how each method addresses muscle restrictions and postural imbalances. The Mézières method, with its holistic, dynamic, and postural approach, may be more effective in trunk rotation by releasing tension and rebalancing posture. On the other hand, isostretching, with its static and muscle targeted approach, could be less effective in this type of movement, as some authors suggest, but can equally contribute to improving other properties such as flexibility and strength [25,26]. These results highlight that the type of stretching influences the outcome, as observed in other studies [12].

The existing literature has demonstrated that both the global postural re-education methods (GPRs), derived from the Mézières method, and isostretching, are effective in treating low back pain [16]. However, the findings of this study suggest that the differences between both approaches may not be so significant, and that both are effective when performed under the supervision of a professional, as shown in the studies by Guastala et al., where patients improved their strength, flexibility, pain, and functionality with both the GPR and isostretching [16].

Regarding the limitations of this study, an important limitation is the relatively small sample size, which could have hindered the detection of significant differences in certain measures.

However, the sample size calculation indicated that a smaller number of individuals was sufficient for the study’s implementation. The small sample size in this study is justified by the highly selective nature of the participant group, consisting of Spanish high-level national rhythmic gymnasts who ranked in top positions in Spain’s National Championships. High-level national athletes represent a specialized population with unique physiological and biomechanical characteristics, making it challenging to recruit large numbers while maintaining homogeneity. Additionally, access to such high-level competitors is limited due to their rigorous training schedules, competition commitments, and the small overall pool of athletes at this level. Despite the limited sample size, the study provides valuable insights into flexibility and mobility adaptations in top-tier gymnasts, as even small groups in elite sports research can yield meaningful conclusions applicable to performance enhancement and injury prevention.

In addition, although efforts were made to ensure the blinding of the evaluators, the nature of the interventions made it impossible to blind the participants, which could have introduced bias.

Electromyography (EMG) analysis was excluded due to the lack of a specialized therapist trained in EMG implementation and interpretation, which made it challenging to assess muscle activation patterns during each movement.

While this study focuses on female gymnasts, future research could explore gender-based differences in training effects, injury risks, and biomechanical adaptations in male rhythmic gymnasts.

Another limitation is the absence of a true control group without intervention. Since both groups received active treatments, this study does not assess the natural progression of low back pain in rhythmic gymnasts or compare these interventions to a non-treatment scenario. While this design allows for a direct comparison of two commonly used postural approaches, future research should consider including a no-intervention control group to better evaluate the absolute effectiveness of these methods.

Additionally, the study was conducted on high-level national rhythmic gymnasts with low back pain, and therefore, the results may not be generalizable to other populations. Furthermore, the duration of the intervention was only 12 weeks, which may not be sufficient to observe changes in certain outcomes.

It would be important to explore the long-term effects of these interventions, increase the sample size, and evaluate different populations. It would also be beneficial to compare these interventions with other treatment techniques, such as core stabilization exercises, balance exercises, or 3D moving platform exercises, which have also demonstrated effectiveness in the treatment of low back pain [25,26,27,28].

## 5. Conclusions

In conclusion, this study provides valuable information about the effects of different treatment approaches for low back pain in high-level national rhythmic gymnasts. Although the Mézières method showed certain advantages in right trunk rotation and left tilt, the results overall suggest that both approaches are effective in the specific context of this population. Future research should address the limitations of this study and explore other treatments and populations.

## Figures and Tables

**Table 1:** Participant’s characteristics.

		Mezieres (*n* = 8)		Isostretching (*n* = 9)		
		Mean	SD	Mean	SD	*p*
Age (years)		13.38	0.92	14.33	4.82	0.589
Height (cm)		157.25	12.85	155.11	6.64	0.667
Weight (kg)		42.00	7.17	45.06	8.97	0.454
Training days per week		4.38	0.52	4.44	0.53	0.788
Training hours per week		14.88	2.47	15.78	3.89	0.582
NRS (cm)		4.94	1.21	5.72	1.48	0.254
Borg (cm)		4.38	1.06	4.00	0.87	0.298
Baseline Trunk movements in grade	Flexion	165.75	15.95	159.56	14.20	0.410
	Extension	82.63	19.20	79.22	12.01	0.663
	Right Rotatation	43.75	12.94	39.67	10.17	0.478
	Left Rotation	34.63	11.99	35.44	8.22	0.870
	Right Inclination	48.88	13.03	42.22	12.06	0.292
	Left Inclination	47.50	10.84	41.33	11.15	0.267

NRS—numeric rating scale; SD—standard deviation; *p*—statistical significance.

**Table 2:** Analysis of covariance between the two study groups.

		Mezieres				Isostretching					Two-Way Repeated Measures ANCOVA					
	*n*	2 Weeks	4 Weeks	6 Weeks	12 Weeks	*n*	2 Weeks	4 Weeks	6 Weeks	12 Weeks	Time		Group		Group ∗ Time	
		Mean (SD)	Mean (SD)	Mean (SD)	Mean (SD)		Mean (SD)	Mean (SD)	Mean (SD)	Mean (SD)	F	*p*	*F*	*p*	*F*	*p*
NRS (cm)	8	5.16 (1.69)	4.33 (1.67)	4.55 (1.50)	4.93 (1.20)	9	3.31 (1.71)	2.43 (1.34)	1.68 (0.70)	1.62 (1.06)	13.007	0.005	10.667	0.000	2.401	0.026
Borg (point)	8	2.87 (1.12)	2.5 (0.92)	1.50 (0.75)	0.50 (0.53)	9	2.11 (1.26)	1.66 (1.00)	1.55 (0.72)	0.66 (0.70)	2.721	0.091	6.473	0.007	2.185	0.143
Flexion (grade)	8	167.12 (12.54)	175.50 (11.96)	175.87 (10.89)	177.12 (14.78)	9	166.55 (18.412)	166.66 (20.34)	158.22 (24.45)	161.77 (23.94)	0.423	0.740	0.400	0.755	1.816	0.198
Extension (grade)	8	88.87 (25.43)	93.87 (27.22)	76.87 (29.43)	78.87 (32.66)	9	92.33 (16.44)	91.00 (13.42)	90.00 (13.48)	89.00 (16.88)	0.198	0.895	0.255	0.856	1.259	0.332
Right Rotation (grade)	8	56.25 (18.28)	47.00 (14.00)	71.75 (19.57)	58.37 (18.30)	9	51.00 (12.27)	48.22 (12.71)	46.22 (10.88)	57.55 (22.00)	1.044	0.409	0.771	0.532	4.619	0.023
Left Rotation (grade)	8	42.75 (14.16)	48.25 (16.30)	66.75 (18.76)	53.50 (12.00)	9	38.22 (8.67)	46.66 (10.80)	46.00 (10.46)	59.77 (28.63)	0.964	0.441	0.99	0.431	3.276	0.059
Right Inclination (grade)	8	59.62 (9.30)	65.00 (12.89)	65.00 (10.39)	63.00 (10.84)	9	61.11 (7.81)	65.77 (7.56)	64.44 (5.63)	63.44 (6.40)	0.439	0.729	0.341	0.796	0.167	0.917
Left Inclination (grade)	8	61.37 (10.48)	63.00 (10.54)	65.87 (6.42)	62.37 (7.03)	9	59.77 (8.00)	61.00 (7.40)	62.88 (4.23)	61.5 (8.05)	14.056	0.000	10.11	0.001	2.119	0.151

NRS—numeric rating scale; SD—standard deviation; F—significant factor; *p*—statistical significance.

## Data Availability

Supporting data will be available upon request.
